# mSphere of Influence: Learning from Nature—Antibody Profiles Important for Protection of Young Infants

**DOI:** 10.1128/mSphere.01021-20

**Published:** 2020-10-14

**Authors:** Esther Ndungo

**Affiliations:** a University of Maryland School of Medicine, Baltimore, Maryland, USA

**Keywords:** antibody profiles, enteric pathogens, maternal-infant immunity, systems serology

## Abstract

Esther Ndungo works in the field of maternal-infant immunity against enteric pathogens. In this mSphere of Influence article, she reflects on how the paper “Fc glycan-mediated regulation of placental antibody transfer” by Jennewein et al. (M. F. Jennewein, I. Goldfarb, S. Dolatshahi, C. Cosgrove, et al., Cell 178:202–215.e14, 2019, https://doi.org/10.1016/j.cell.2019.05.044) impressed upon her the value of thinking “outside the box” and looking to nature to guide her research.

## COMMENTARY

The neonatal stage, during which the immune system is developing, leaves the infant vulnerable to infectious threats. Nature provides a helping hand through placental transfer of antibodies from mother to child during pregnancy, and through immune components in breast milk after birth. Vaccinating mothers during pregnancy is therefore a promising strategy to provide passive immunity against infectious diseases during the first few months of life. The particular mechanisms involved in transfer of immune defenses from mother to infant are incompletely understood. Descriptions of differences in neonatal Fc receptor (FcRn) binding and the relative transfer of IgG subclasses are not sufficient to explain the process, since antibodies with different antigen specificities are not transferred equally (1). In addition, the functional capacity of the transferred antibodies against infectious agents remains to be understood. Jennewein et al. (2) tackled this question by performing a systems serology analysis of antibodies in maternal and cord blood pairs to identify functional and structural features of vaccine-induced antibodies “sieved” by the placenta. Systems serology is a novel, high-dimensional approach that defines biophysical antibody profiles (Fc receptor [FcR] binding, subclasses, glycan analysis) as well as antibody-dependent Fc-effector functions (complement binding, phagocytosis, cytotoxicity, chemokine/cytokine secretion). This is augmented by computational methods to mine the vast amount of data obtained to decipher features important for specific functions.

The authors identified maternal-infant transfer of antibodies with distinct functional features specific to four pertussis antigens included in the tetanus, diphtheria, and pertussis (Tdap) vaccine: pertactin (PTN), filamentous hemagglutinin (FHA), fimbriae 2/3 (FIM), and pertussis toxin (PTX). Functional activity was determined by examining antibody-enabled phagocytosis of antigen-coated beads by innate immune cells (monocytes or neutrophils). The transfer of these antibodies from mother to infant was heterogeneous across antigens with no clear trend. The authors also measured transfer of natural-killer (NK) cell-activating antibodies (through increases in CD107a, gamma interferon [IFN-γ]), and macrophage inflammatory protein-1β [MIP-1β]). In contrast to innate phagocytic activity, a consistently higher transfer efficiency of NK cell-activating antibodies against all four antigens was observed. Intriguingly, this preferential transfer of NK cell-activating antibodies was true not only for pertussis antibodies but also for antibodies against respiratory syncytial virus (RSV), influenza virus, and measles virus, consistent with a broad, as opposed to antigen-specific, mechanism.

The authors then performed a step-by-step dissection of the biophysical Fc characteristics of antibodies in maternal and cord blood serum. Subclass analysis confirmed higher transfer efficiencies of IgG1, which is well established. However, IgG1 transfer ratios were not equal across antigen specificities. Next was an assessment of the Fc glycosylation profile, which is known to influence strength of antibody binding to FcR, thereby regulating antibody effector function. Jennewein et al.’s glycan analysis revealed that placental transfer favored galactosylated and sialylated antibodies. This is of interest as galactosylation has been correlated with a low inflammatory state. Whether this is a global adaptation resulting from the state of pregnancy *per se*, or an evolutionary leverage to facilitate placental transfer and benefit the offspring, remains to be determined. Galactosylation improved binding to the FcRn, which is known to transport antibodies through the placenta in a pH-dependent manner. Binding to FcγIIIa was also improved; this FcR has not been described as a transplacental conduit, but the authors speculated that it may work synergistically with FcRn to facilitate antibody transfer. These results were corroborated by Martinez et al., who also reported a preference for FcγIIa and FcγIIIa binding in cord blood (3). Computational analysis, employing partial least squares regression (PLSR) models, confirmed a skewing/enrichment of the cord blood antibody profile toward NK cell activation, Fc glycosylation, and Fc receptor binding as key determinants of placental antibody transfer. Taken together, this work demonstrated the value of systems serology as a powerful data mining tool to uncover unique properties of humoral responses to infection, particularly in special populations.

The observation that placental transfer was systematically (not randomly) biased toward antibodies that activate NK cells stood out to me. As nature would have it, placental “sieving” favors antibodies that activate the most functional innate cells in the context of the immature neonatal immune system. I was intrigued by these unexpected findings, which emphasized the importance of observing and learning from nature to guide my research. My work is aimed at developing vaccines against enteric infections that would protect children, especially very young infants. As we seek to improve the efficacy of vaccines for this vulnerable population, a careful analysis of antibody characteristics and functions that are important for protection in children during the first months of life and after maternal immunity wanes would be invaluable.

I was also impressed that the authors went beyond what was already known about antibody transfer to venture into novel features and functionalities, employing computational models to integrate the data and better interpret the maternal-infant humoral immune profile. For my research, this means thinking broadly and going beyond what is known about natural immunity, vaccination, and the role of antibodies and not overlooking other unique attributes, specificities, or functions that have not yet been implicated but may prove to be important for preventing infections in children. I am inspired to use systems serology to investigate the effect of the gut environment (microbiome or disease state) and also external factors (nutrition and access to resources) on vaccine-induced antibody structure/function in settings which bear the greatest burden of enteric disease but where prophylactic strategies have not always been effective (4). As an example, Martinez et al. used systems serology to show that HIV-infected mothers had impaired placental IgG transfer that could be attributed to modified Fc glycan structures (3).

Since publication of this paper, the systems serology suite of assays has been expanded to test for functionality of different immune cell types (5), increasing its versatility. The technology has been used to characterize humoral responses to diverse pathogens, e.g., HIV (6), Mycobacterium tuberculosis (7), and Ebola virus (8), illustrating its potential to be leveraged against new and emerging pathogens. As we anticipate a vaccine against COVID-19, for example, what we have learned from systems serology could guide us in the design of candidates that would generate effective humoral immunity in pregnant women, children, or the most vulnerable elderly population. This publication illuminated how much is yet to be learned about antibody protection in children but that I need only look to nature, which does it best.
